# Mental Disorders among Children and Adolescents Admitted to a French Psychiatric Emergency Service

**DOI:** 10.1155/2013/651530

**Published:** 2013-01-28

**Authors:** Laurent Boyer, Jean-Marc Henry, Jean-Claude Samuelian, Raoul Belzeaux, Pascal Auquier, Christophe Lancon, David Da Fonseca

**Affiliations:** ^1^EA 3279, Self-Perceived Health Assessment Research Unit, Faculté de Médicine, Université Aix-Marseille, 13005 Marseille, France; ^2^Department of Psychiatry, AP-HM Hôpital de la conception, 13855 Marseille, France; ^3^Department of Psychiatry, AP-HM Hôpital Sainte-Marguerite, 13274 Marseille, France; ^4^Department of Pedosychiatry, AP-HM Hôpital Sainte-Marguerite & Salvator, 13274 Marseille, France

## Abstract

The aim of this study was to describe the characteristics of children and adolescents admitted to the psychiatric emergency department (ED) of a French public teaching hospital over a six-year study period (2001–2006). Data for all episodes of care in the psychiatric ED from January 1, 2001, to December 31, 2006, delivered to adolescents aged less than 18 years were retrospectively analyzed. During the six-year study period, 335 episodes of care in the psychiatric ED were experienced by 264 different adolescents. They accounted for 2.0% of the 16,754 care episodes of the ED; 164 patients (62.1) were female and the average age was 16.5 (SD = 1.6). The neurotic, stress-related, and somatoform disorders were the most frequent (25.4%) and concerned mainly anxiety disorders (15.2%). The frequency of the absence of psychiatric diagnosis (22.7%) was high. A total of 48 children and adolescents (18.2%) benefited from more than one episode of care. Several factors were associated to a higher number of visits to the ED: substance use, schizophrenia, disorders of adult personality and behaviour, disorders occurring in childhood and adolescence, and dual diagnosis. In conclusion, mental health disorders in children and adolescents are a serious problem associated with several potentially modifiable factors.

## 1. Introduction

Mental health disorders in children and adolescents are a growing public health problem in developed countries [1, 2]. The prevalence of these disorders in France and in the United States has been estimated to be at least 12.5% and 20%, respectively [3–5]. According to the World Health Organization in 2001, mental health problems among children and adolescents have increased in recent years and are predicted to increase by 50% by the year 2020 [6]. Management of these mental health care needs remains challenging. A special effort should be made to improve access to needed mental health care services [1]. Mental health disorders among children and adolescents are often unrecognised and untreated [4, 7]. Once identified, only 1 in 5 children and adolescents with mental health problems receive mental health treatment [8–10], and the absence of health insurance coverage plays a critical role in access to mental health care services [11]. Given the high proportion of unmet mental health care needs among children and adolescents, psychiatric emergency departments (PEDs) often serve as the entry point into the mental health system [7, 10]. Developing a better knowledge and understanding of the characteristics of children and adolescents presenting to the PED could lead to improvements in the delivery of mental health care in both emergency and outpatient psychiatric settings [12]. 

Mental health disorders in children and adolescents have received significant attention in the past several years. However, few studies have examined the use of psychiatric emergency services by children and adolescents, although emergency services can be considered an interesting place of observation and intervention [2, 13]. Most studies have been conducted in countries in which children and adolescents do not have universal health coverage. The evaluation and management of children and adolescents in the French health care system, by contrast, especially in psychiatric emergency services, may contribute to a particularly valuable analysis of their needs. The French health care system combines near-universal coverage with a public-private mix of hospital care and ambulatory care, as well as a higher volume of service provision than the American health care system [14]. 

The aim of this study was to describe the characteristics (demographic, clinical, and management) of children and adolescents admitted to the PED of a French public teaching hospital over a six-year study period (2001–2006).

## 2. Methods

### 2.1. Setting

Marseille, located in the south-eastern part of France, is the second largest city in France, with a population of nearly one million inhabitants. This study was conducted in the PED of a large French public teaching hospital in Marseille (Assistance Publique—Hôpitaux de Marseille) that is responsible for evaluating and treating persons (children, adolescents, and adults) with psychiatric disorders. Care was provided by general psychiatrists. Annual visits to this ED number approximately 3,000. It provides services to 95% of the individuals living in Middletown, Marseille, who present to psychiatric emergencies 15. The ED also guarantees free medical service 24 hours a day, seven days a week, to natives and immigrants, regardless of their legal, social, or economic status. Of note, our hospital has also 2 other emergency departments for paediatric and general cares. If necessary, patients with mental health problems are transferred from these emergency departments to the PED. Finally, a child psychiatrist from local child and adolescent psychiatric services has to be on-call 24 hours a day for the PED.

### 2.2. Population

Data for all episodes of care in the PED from January 1, 2001, to December 31, 2006, delivered on children and adolescents aged less than 18 years were retrospectively analyzed.

### 2.3. Data Collection

The study involved a retrospective review of administrative and medical databases from the PED. For each episode of care, data on the patient's demographic, clinical, and management characteristics were available. The French National Commission for Data Protection (CNIL) approved this study. Founded on January 6, 1978, the CNIL is an independent administrative authority protecting privacy and personal data [15]. Because the project involved the retrospective analysis of anonymous administrative data sets—patient names were replaced by a unique identification number—informed consent was not necessary. Principles outlined in the Declaration of Helsinki were followed [16].

### 2.4. Units of Analysis and Definition of Parameters

Because some individuals received multiple episodes of care during the study period, two analysis sets were used: episodes of emergency care and individual patients. If readmission rates varied significantly between patients, the exclusive use of episode data could bias the results.

For emergency care episodes, the following data were examined: reasons for referral, referral source (self-referral, referred by the family, referred by a health care professional, referred by a somatic ED, or referred by a nonhealth care professional), and nature of therapeutic crisis management (consultation or hospitalisation).

For individual patients, demographic and clinical data were collected. Demographic information consisted of age (defined as the mean age during the various contacts) and gender. Clinical information included psychiatric diagnosis. Because patients may present with more than one psychiatric diagnosis in one or several care episodes, we described the diagnostic characteristics only for the individual patients. Diagnoses were established by a senior general psychiatrist based on the *ICD-10 sections* [17]. Dual diagnosis was defined as the co-occurrence of a mental disorder (any) and a substance-related disorder [18]. Other data regarding the PED analyzed during the study period included the number of emergency care episodes.

### 2.5. Statistical Analysis

The demographic, clinical, and management characteristics of children and adolescents were described first for care episodes and then for individual patients. Sample characteristics were detailed using means for quantitative variables or frequencies for qualitative variables and their 95% confidence intervals.

The demographic, clinical, and management characteristics were also compared using Student's *t*-test for continuous variables and chi-squared analysis for categorical variables according to gender, age (</≥16 years), and return to the ED (yes/no). The comparisons were made only for individual patients. All tests were two tailed, and the alpha value was set at *P* < .05. Data were analyzed using the SPSS, version 18.0, software package.

## 3. Results

During the six-year study period (2001–2006), 335 episodes of care in the PED were experienced by 264 different children and adolescents. They accounted for 2.0% of the 16,754 care episodes in the ED.

### 3.1. Episodes of Emergency Care (*N* = 335)

As shown in Table 1, the most frequent referral sources for admission were the somatic ED (34.4%), the family (30.2%), or the patient (12.3%). Anxiety, disruptive behavior, and suicide attempts were the most frequent reasons for referral. Approximately 87.9% of suicide attempts were by self-poisoning. Agitation, aggressive behaviour, psychotic symptoms, depressive symptoms, alcohol and substance use disorders, and relational problems were less frequent, between 3.6 and 7.5%. Children and adolescents were hospitalised in 41.3% of episodes.

### 3.2. Individual Patients (*N* = 264)

As shown in Table 2, 164 patients (62.1%) were female. The average age was 16.5 (SD = 1.6); 3.8% were less than 14 years old, 48% were between 14 and 16 years old, and 78% were older than 16 years of age. The neurotic, stress-related, and somatoform disorders (*ICD*-10 section F4) were the most frequent (25.4%) and included mainly anxiety disorders (15.2%). Disorders of adult personality and behavior (*ICD*-10 section F6) represented 16.7%, while specific childhood diagnoses (*ICD*-10 sections F8, F9) represented 0.8% and 12.8%, respectively. Mood (affective) disorders (*ICD*-10 section F3) represented 14.0% and included mild or moderate depressive episodes in 9.1%. Schizophrenia (*ICD*-10 section F2) and substance use disorders (ICD-10 section F1 6.8% and dual diagnosis 3.8%) were reported equally at 10.6%. The frequency of nonpsychiatric diagnosis (22.7%) was relatively high. The chief complaint for these patients with nonpsychiatric diagnoses was disruptive behaviour. A total of 48 children and adolescents (18.2%) received more than one episode of care.

As shown in Table 3, there were no statistically significant differences in demographic, clinical, and management characteristics according to age (< or ≥16 y) or gender except for cases of schizophrenia, which was diagnosed more frequently in the male group. On the contrary, several factors were associated with a higher number of visits to the ED: substance use, schizophrenia, disorders of adult personality and behaviour, disorders occurring in childhood and adolescence, and dual diagnoses.

## 4. Discussion

This study revealed several important findings related to children and adolescents who present to a PED. One of this study's strengths was that it investigated activity over a 6-year period in a psychiatric emergency ward in a health care system characterised by near-universal access. This study is one of the first conducted in this type of health care system. Considering this characteristic, several hypotheses must be discussed and clarified.

### 4.1. Characteristics of Children and Adolescents

First, the study involved primarily older adolescents (78% were 16-17 years old), a proportion that is higher than in previous studies [10]. One related hypothesis could be that the specific needs of older adolescents are relatively neglected compared to younger children. These specific needs should be addressed along with the general needs of younger children.

Second, in line with other studies, suicide attempts were the main reason for referral to the PED (24.5%). However, the percentage of individuals presenting with this complaint was lower than in other studies [9, 19]. Suicidal ideation was the chief complaint in 39% of patients in the study by Santiago et al. [19] and 47% in the study by Grupp-Phelan et al. [9]. One explanation for the discrepancy between this study and others could be that a portion of individuals with suicide attempts are referred to the somatic ED and not to the PED. All adolescents with possible suicide attempts should receive a comprehensive mental health and psychosocial assessments [20]. Strategies for engaging high-risk families into mental health services might include psychiatric ED-based suicide interventions, and for this process, a better collaboration between the somatic and PED should be developed. Studies show that half of the adolescents seen for suicidal behavior in the PED are never referred for treatment, and, even if referred, the compliance with treatment is low [21].

Third, an interesting finding is the discordance between the proportion of referrals for depressive symptoms (5.4%) and the proportion of psychiatric diagnoses of depression (10.2%). Depression in adolescents is often associated with a varied presentation of symptoms. This variability can explain why the reason for referral is not systematically the depressive symptoms. This study suggests that the ED plays an important role in identifying depression.

Fourth, even if depression was one of the main diagnoses in this study, the result is lower than in other studies [9]. In the same way, aggressive behavior only concerned 6.9% of children and adolescents contrary to previous investigations [10, 19, 22]. Aggressive or violent behavior concerned 42% of patients in the study by Santiago et al. [19] and 37% in the study by Edelsohn et al. [10]. One explanation for this difference may be that this population is not the same as those in other studies. We can hypothesise that the universal coverage and the absence of economic barriers to care likely improve access to outpatient psychiatric services and, consequently, that depression or aggressive behavior is less of an unmet mental health care need among children and adolescents in this country.

Sixth, the frequency of disorders of adult personality and behavior (ICD-10 section F6) seems particularly high (16.7%). The onset of these patterns of behavior can typically be traced back to late adolescence, the beginning of adulthood, and, in rare instances, childhood [17]. It is therefore unlikely that a diagnosis of personality disorder will be appropriate before the age of 16 or 17 years. The majority of patients with disorders of adult personality and behavior were older than 16 years old (38/44 = 86%), a finding that is not in contradiction with the definition of disorders of adult personality and behaviour. Youths with cluster B personality disorders are well known to be overrepresented in EDs requiring psychiatric assessment, especially the repetitive ED users.

Finally, the proportion of nonpsychiatric diagnoses is also high (22.7%) and may include nonpsychiatric emergencies and potentially inappropriate visits. This hypothesis has been suggested in several studies. In the study of Soto et al. [23], over one-third of ED visits were inappropriate. The reasons that patients and families seek care in the PED instead of as outpatients should be examined directly in future studies to identify and address the barriers towards the appropriate use of mental health services. Another explanation for this finding is the inaccurate diagnoses. As suggested previously, the fact that general psychiatrists made the diagnoses may be an explanation for this finding.

### 4.2. Use of the PED

In this study, children and adolescents represented 2.0% of the patients who visited the PED during the six-year study period. This finding is similar to that of other studies of the somatic ED, but lower than the proportion seen in studies of the PED [2, 24]. One hypothesis is that with a common PED for adults and children, admitting young people to a general PED (mainly occupied by adults) can be problematic. The presence of adults with psychiatric disorders (many potentially severe) might prevent children and adolescents and their families from visiting the ED.

The parents of the children or adolescents were the most likely referral source for admission (42.3%). This result is consistent with previous studies [9, 23] and may indicate that children and adolescents are not receiving needed mental health services and that the ED can be considered as the final safety net for people whose needs are not met elsewhere in the health care system [10]. The ED functions both as a crisis service and as a “backdoor” alternative when community-based services cannot meet the mental health needs of children and adolescents. Special attention should be given to parents. Specific programmes may help them and may decrease parental psychological distress. For example, the study of Power et al. [25] showed that a support programme for parents of young people with suicidal behavior was a promising development. This study indicated positive results for the parents who completed the programme. Parents had lower levels of psychological distress and higher levels of satisfaction [25]. The needs of children and adolescents should also be taken into account in assessments of health care needs [26, 27]. The results of the study by Rajmil et al. [27] showed that children and adolescents are a suitable source for the self-assessment of their health-related quality of life. The reasons that patients and parents seek the ED instead of outpatient care should be examined in future studies to identify and address barriers to care and unmet needs.

Children and adolescents who use the ED repeatedly represent a significant proportion of youth psychiatric emergencies (18.2%). This study identified factors associated with ED recidivism: substance use and dual diagnosis, schizophrenia, disorders of adult personality and behaviour, and disorders occurring in childhood and adolescence. First, these results suggest that the ED is being used as part of a continuum of care for patients already in treatment, particularly those displaying disruptive behavior (schizophrenia and disorders occurring in childhood and adolescence). For Christodulu et al. [28], repeat users are less compliant with outpatient followup. Second, substance use disorders are well known as factors associated with recidivism in the ED [29, 30]. Although these factors are well known, health systems have difficulty taking them into account. The French health care system tends to focus on either mental health or substance abuse treatment services [29]. This result can be an indication of insufficient and fragmented dual diagnosis services. Finally, recidivism was also associated with the presence of disorders of adult personality and behaviour and is thus an important issue to consider.

### 4.3. Limitations

This study has several limitations. First, the data source was an administrative database. Several sociodemographic characteristics, for example, ethnicity, were not collected due to the French context. Diagnoses made in an emergency context by a large panel of general psychiatrists may not be as accurate as those made using diagnostic research instruments and structured interviews. Moreover, general psychiatrists in a PED have not been specifically trained in assessing and diagnosing child and adolescent psychiatric disorders. Additionally, information regarding illness severity and psychiatric comorbidities was not always available. In particular, psychiatric comorbidities which are very frequent in children and adolescences should be explored in future studies. Finally, nonpsychiatric diagnoses were imprecise in our database, and this issue clearly merits more attention in the future.

Second, a lack of power can explain the absence of statistical differences by age or gender in the third part of this analysis. These results must be confirmed with a larger sample. Finally, because this study was conducted in one large French teaching hospital, these findings may not be extrapolated to all of the other hospitals in France and to health care systems without universal coverage. However, these results are useful for monitoring policy and practice changes in a community because this study was conducted in a domain that is poorly understood [2]. Additional research is required if we are to understand how the ED can best assist communities in developing systems of care. Studies with different methodology, especially using prospective design, should be conducted to test these hypotheses. A more comprehensive investigation into the experience of the psychiatric patient, using qualitative methods, could provide important information.

## 5. Implications for Behavioral Health

The universal coverage and the absence of economic barriers to care likely facilitate and improve the access to outpatient psychiatric services. A large proportion of the health care needs are likely already met by French outpatient psychiatric services, and this access to care can explain why only 2% of visits concern children and adolescents. As a consequence, the psychiatric EDs are mainly organised to manage adults. Indeed, only general psychiatrists work in the French psychiatric ED. The question that remains is as follows: how do we determine whether the psychiatric EDs should focus a part of their resource allocation and potential interventions on children and adolescents up to 18 years old. In the same way, the question of creating a paediatric psychiatric ED for a relatively low proportion of children and adolescents with mental health disorders should also be discussed. In our context, it seems however more adapted to develop more effective communication between local child and adolescent psychiatric services to ensure timely admissions and discharge with effective followup.

In conclusion, universal coverage systems face a real problem associated with the constraints of limited resources, and they must select priorities between various health interventions or programs. Economic cost-effectiveness analysis is often used to set priorities for resource allocation among available health interventions. Specific resource allocation and potential intervention for only 2% of visits for children and adolescents are probably not cost-effective. However, because of their empirical and theoretical limitations, the results of economic cost-effectiveness analyses should be but one element in the priority setting in health care systems, which should also be informed by explicit debates about societal and ethical preferences.

## Figures and Tables

**Table 1:** Characteristics associated with episodes of care provided by a psychiatric emergency department of a French public hospital (2001–2006).

Episode measure (*N* = 335)	*N*	%	95% CI^a^
Reason for referral			
Agitation	25	7.5	[5.7; 9.2]
Anxiety	76	22.7	[19.9; 25.5]
Aggressive behaviour	23	6.9	[5.2; 8.6]
Disruptive behaviour	73	21.8	[19.0; 24.5]
Mental confusion	1	0.0	[0.0; 0.7]
Psychotic symptoms	12	3.6	[2.3; 4.8]
Depressive symptoms	18	5.4	[3.9; 6.9]
Manic symptoms	0	—	—
Suicide attempt	82	24.5	[21.6; 27.3]
Cognitive disorder	0	—	—
Alcohol use related	8	2.4	[1.4; 3.4]
Drug use related	7	2.1	[1.1; 3.0]
Somatic problem	6	1.8	[0.9; 2.7]
Relational problem	20	6.0	[4.4; 7.6]
Social problem	6	1.8	[0.9; 2.7]
Crisis management: referral source for admission			
Self-referral	41	12.3	[10.1; 14.5]
Family	101	30.2	[27.2; 33.3]
Health care professionals	38	11.4	[9.3; 13.5]
Somatic emergency department	115	34.4	[31.3; 37.6]
Nonhealth care professional	39	11.7	[9.5; 13.8]
Crisis management: consultation or hospitalisation			
Consultation	203	58.5	[55.2; 61.8]
Hospitalisation	132	41.3	[38.0; 44.6]

^a^95% CI: 95% confidence interval.

**Table 2:** Characteristics of child and adolescent patients in a psychiatric emergency department of a French public hospital (2001–2006).

Characteristics (*N* = 264)	*N*	%	95% CI^a^
*Demographic *			
Age (mean ± SD years)	16.5	1.6	[16.4; 16.6]
Age class			
<14	10	3.8	[2.5; 5.1]
[14–16[	48	18.2	[15.6; 20.8]
[16–18[	206	78.0	[75.3; 80.8]
Female	164	62.1	[58.1; 66.1]
*Psychiatric diagnosis *			
Mental and behavioural disorders resulting from psychoactive substance use (ICD-10 section F1)	18	6.8	[4.7; 8.9]
Alcohol	6	2.3	[1.0; 3.5]
Cocaine	2	0.8	[0.0; 1.5]
Cannabinoids	6	2.3	[1.0; 3.5]
Schizophrenia and schizotypal and delusional disorders (ICD-10 section F2)	28	10.6	[8.1; 13.1]
Mood (affective) disorders (ICD-10 section F3)	37	14.0	[11.2; 16.9]
Mild or moderate depressive episode	24	9.1	[6.7; 11.5]
Severe depressive episode	3	1.1	[0.3; 2.0]
Neurotic, stress-related, and somatoform disorders (ICD-10 section F4)	67	25.4	[21.8; 29.0]
Anxiety disorder	40	15.2	[12.2; 18.1]
Reaction to severe stress	12	4.5	[2.8; 6.3]
adjustment disorder	8	3.0	[1.6; 4.4]
Behavioural syndromes associated with physiological disturbances and physical factors (ICD-10 section F5)	9	3.4	[1.9; 4.9]
Eating disorder	8	3.0	[1.6; 4.4]
Disorders of adult personality and behavior (ICD-10 section F6)	44	16.7	[13.6; 19.7]
Dissocial personality disorder	12	4.5	[2.8; 6.3]
Emotionally unstable personality disorder	10	3.8	[2.2; 5.4]
Mental retardation (ICD-10 section F7)	3	1.1	[0.3; 2.0]
Disorders of psychological development (ICD-10 section F8)	2	0.8	[0.0; 1.5]
Behavioural and emotional disorders with onset during occurrence in childhood and adolescence (ICD-10 section F9)	34	12.9	[1.3; 4.0]
Conduct disorders	22	8.3	[6.1; 10.6]
Mixed disorders of conduct and emotions	9	3.4	[1.9; 4.9]
Dual diagnosis^b^	10	3.8	[2.2; 5.4]
*Absence of psychiatric diagnosis *	60	22.7	[19.9; 25.5]
*Number of admissions, 2001–2006 *			
1	216	81.8	[78.6; 85.0]
+2	48	18.2	[15.0; 21.4]

^a^95% CI: 95% confidence interval.

^b^Substance use abuse plus any other diagnosis listed previously.

**Table 3:** Characteristics of child and adolescent patients in a psychiatric emergency department of a French public hospital (2001–2006) by gender, age and number of admissions.

	Gender					Age					Number of admissions, 2001–2006				
Characteristics (*N* = 264)	Female		Male			<16		≥16			1		+2		
	*N* = 164		*N* = 100		*P *	*N* = 58		*N* = 206		*P *	*N* = 216		*N* = 48		*P *
	*N *	%	*N *	%		*N *	%	*N *	%		*N *	%	*N *	%	

*Demographic *															
Age (Mean ± SD years)	16.7	1.2	16.3	2.1	0.12	—	—	—	—	—	16.5	1.7	16.7	1.3	0.32
Female	—	—	—	—	—	35	60.3	129	62.6	0.75	140	64.8	26	54.0	0.05
*Psychiatric diagnosis *															
Mental and behavioural disorders resulting from psychoactive substance use	8	4.9	10	10.0	0.10	1	1.7	17	8.3	0.08	11	5.1	7	14.6	**0.02**
Schizophrenia and schizotypal and delusional disorders	11	6.7	17	17.0	**0.01**	5	8.6	23	11.2	0.58	19	8.8	9	18.8	**0.04**
Mood (affective) disorders	11	11.0	26	15.9	0.27	9	15.5	28	13.6	0.71	29	13.4	8	16.7	0.56
Neurotic, stress-related, and somatoform disorders	44	26.8	23	23.0	0.49	9	15.5	58	28.2	0.05	54	25.0	13	27.1	0.76
Behavioural syndromes associated with physiological disturbances and physical factors	8	4.9	1	1.0	0.16	3	5.2	6	2.9	0.40	6	2.8	3	6.3	0.21
Disorders of adult personality and behaviour	26	15.9	18	18.0	0.65	6	10.3	38	18.4	0.14	27	12.5	17	35.4	**<0.01**
Mental retardation	1	0.6	2	2.0	0.56	2	3.4	1	0.5	0.06	1	0.5	2	4.2	0.09
Disorders of psychological development	1	0.6	1	1.0	1.00	1	1.7	1	0.5	0.33	2	0.9	0	0.0	1.00
Behavioural and emotional disorders with onset during occurring in childhood and adolescence	23	14.0	11	11.0	0.48	11	19.0	23	11.2	0.12	21	9.7	13	27.1	**0.01**
Dual diagnosis	5	3.5	5	5.0	0.51	0	—	10	4.9	0.09	3	1.4	7	14.6	**<0.01**
*Absence of psychiatric diagnosis *	41	25.0	19	19.0	0.26	17	29.3	43	20.9	0.18	57	26.4	3	6.3	**<0.01**
*Number of admissions, 2001–2006 *															
1	140	85.4	76	76.0	0.05	49	84.5	167	81.1	0.55	—	—	—	—	—
+2	24	14.6	24	24.0		9	15.5	39	18.9						
